# Immediate Effects of (Simulated) Age-Related Hearing Loss on Cognitive Processing and Performance for the Backward-Digit-Span Task

**DOI:** 10.3389/fnagi.2022.912746

**Published:** 2022-10-13

**Authors:** Christian Füllgrabe, Ozan Cem Öztürk

**Affiliations:** ^1^Ear Institute, University College London, London, United Kingdom; ^2^School of Sport, Exercise and Health Sciences, Loughborough University, Loughborough, United Kingdom

**Keywords:** backward digit span, cognitive assessment, impairment simulation, age-related hearing loss, short-term memory, working memory

## Abstract

The recall of auditorily presented sequences of digits in reverse order (also known as the Backward Digit Span, BDS) is considered to reflect a person’s information storage and processing abilities which have been linked to speech-in-noise intelligibility. However, especially in aging research and audiology, persons who are administered the BDS task are often affected by hearing loss (HL). If uncorrected, HL can have immediate assessment-format-related effects on cognitive-test performance and can result, in the long term, in neuroplastic changes impacting cognitive functioning. In the present study, an impairment-simulation approach, mimicking mild-to-moderate age-related HLs typical for persons aged 65, 75, and 85 years, was used in 19 young normal-hearing participants to evaluate the impact of HL on cognitive performance and the cognitive processes probed by the BDS task. Participants completed the BDS task in several listening conditions, as well as several commonly used visual tests of short-term and working memory. The results indicated that BDS performance was impaired by a simulated HL representing that of persons aged 75 years and above. In the normal-hearing condition, BDS performance correlated positively with both performance on tests of short-term memory and performance on tests of working memory. In the listening condition simulating moderate HL (as experienced by the average 85-year-old person), BDS performance only correlated with performance on working-memory tests. In conclusion, simulated (and, by extrapolation, actual) age-related HL negatively affects cognitive-test performance and may change the composition of the cognitive processes associated with the completion of a cognitive task.

## Introduction

Traditionally, cognitive assessments are employed in the study of normal and pathological cognitive development and aging (Ford et al., 2012; Weintraub et al., 2013; Salthouse, 2019) and for the screening of neurological and behavioral functions and clinical diagnosis (Brandt, 1991; Nasreddine et al., 2005; Larner, 2017). In recent years, cognitive abilities have also been assessed with increasing frequency in research in speech and hearing sciences and as part of the clinical practice in hearing health care (HHC; Pichora-Fuller and Singh, 2006; Valente et al., 2006; Füllgrabe and Rosen, 2016). Conducting cognitive tests fulfills various purposes for the hearing scientist and the HHC professional. For example, cognitive screening allows the enforcement of cognitive inclusion or exclusion criteria for and the adjustment of hearing and speech-identification assessments on the basis of the cognitive status of the participants (Füllgrabe et al., 2018; Bott et al., 2019; British Society of Audiology, 2021). In addition, cognitive profiling can further the understanding of individual variability in (un)aided speech identification (Humes et al., 2013; Füllgrabe et al., 2015; Nuesse et al., 2018), and help predict benefits associated with different hearing-aid processing features (Lunner et al., 2009; Neher, 2014; Ohlenforst et al., 2016) as part of a more individualized auditory rehabilitation (Kiessling et al., 2003; Kricos, 2006; Pichora-Fuller and Singh, 2006). It is also being debated whether to expand the scope of practice of the HHC professional to include routine cognitive screening of older adults, with the aim of detecting cognitive impairment, providing counselling, and, if indicated by the results, referring to a mental-healthcare professional for diagnostic evaluation (Armero et al., 2017; American Speech-Language-Hearing Association, 2018; Beck et al., 2018).

However, the generalized use of cognitive tests is not viewed uncritically due to potential intrinsic biases, such as cultural, socioeconomic, and educational factors (Parker and Philp, 2004; Crane et al., 2008; Reynolds and Suzuki, 2012). It also has been acknowledged that, in the older population, cognitive performance may be detrimentally affected by the interaction between age-related changes in peripheral sensory functions and the presentation format of the cognitive assessment (Schaie, 2004; Wingfield et al., 2005; Ben-David et al., 2018). Indeed, there is converging evidence that older people with age-related sensorineural hearing loss (HL) score significantly lower than age-matched normal-hearing (NH) controls on a variety of cognitive tasks (McCoy et al., 2005; Dupuis et al., 2015). Yet, the observation of a deficit in cognitive performance in those individuals does not demonstrate the existence of assessment-related auditory biases, as a reduction in cognitive functioning could also be caused by permanent neuroplastic brain changes in response to prolonged sensory deprivation (Schneider and Pichora-Fuller, 2000; Griffiths et al., 2020). In the latter case, the cognitive deficits do indeed have an auditory origin but are not necessarily related to the presentation format of the cognitive assessment.

Supporting evidence that HL has an immediate deleterious effect on cognitive performance due to the auditory format of the cognitive test employed comes from simulation studies in which auditory deficits are temporarily induced in young NH adults for the duration of the cognitive assessment. In most cases, however, only the effect of a reduction in audibility was investigated (Lindenberger et al., 2001; Jorgensen et al., 2016; Gaeta et al., 2019), and, thus, the true size of the auditory bias was likely underestimated. To mimic the impact of a wider range of perceptual consequences of age-related HL (ARHL) on cognitive-test performance, Füllgrabe (2020a) used an HL simulator mimicking not only elevated hearing thresholds but also reduced frequency selectivity and loudness recruitment. In this study, 56 young NH participants were randomly assigned to one of two listening conditions to perform different memory tasks. Compared to the control condition using unprocessed test stimuli, the simulated-HL condition yielded significantly worse performance on all cognitive tasks. However, due to the use of a between-subject design, a possible sampling bias cannot be ruled out.

To corroborate the assumption that cognitive assessments are prone to auditory biases, the main aim of the present study was to replicate the findings of Füllgrabe (2020a), this time using a within-subject design, in which each participant is tested in all listening conditions that were extended to less and more severe levels of simulated HL compared to the study of Füllgrabe. It was hypothesized that memory performance would be worse in listening conditions simulating HL.

A secondary aim of the present study was to explore whether the mental processes probed by a given cognitive task change as a function of the individual characteristics of the person being assessed (e.g., age, hearing status). Using a cognitive task frequently administered in hearing research in conjunction with measures of speech-in-noise identification (Gieseler et al., 2017; Hillyer et al., 2019; Kamerer et al., 2019), St Clair-Thompson (2010) reported evidence that the backward-digit-span (BDS) task can be described as a measure of working memory (WM) in children, while it probes short-term memory (STM) in adults. To investigate the impact of simulated HL on the cognitive processes at work during the completion of the BDS task, participants were administered the BDS task as well as tests of STM and WM, with the aim of computing the correlational strength between performances on the different tasks. It was hypothesized that performance on the BDS task would be differentially associated with performances on the STM and WM tasks in the NH and simulated-HL conditions.

## Materials and Methods

### Participants

Nineteen (nine females) native-English-speaking volunteers were recruited from the undergraduate student population of Loughborough University (United Kingdom). Their ages ranged from 20 to 25 years (mean age = 22.3 years; standard deviation = 1.4). All participants had normal (i.e., ≤ 20 dB Hearing Level) audiometric thresholds in the test (i.e., right) ear at octave frequencies between 0.25 and 4 kHz, assessed following the procedure recommended by the British Society of Audiology (2018) and using standard calibrated audiometric equipment. They also had self-reported normal or corrected-to-normal vision.

### Stimuli and Procedure

#### General Procedure

Participants attended a single test session lasting approximately 90 min. After providing demographic and visual-acuity information, and passing the audiometric screen for normal hearing sensitivity, each participant completed five memory tasks: first, two STM tasks, followed by the BDS task, and, finally, two WM tasks. Short breaks were enforced before and after the BDS task to reduce fatigue. STM and WM tasks were presented visually and in a nearly counterbalanced order across participants. The BDS task was presented auditorily in four listening conditions: first in the “NH condition”, and then in three “simulated-HL conditions” presented in a nearly counterbalanced order across participants.

Following the administration of the BDS task, the ability to understand the stimuli in the most severe simulated-HL condition used in the present study (i.e., a moderate HL as experienced by the average 85-year-old person; see Section “Backward-Digit-Span Task and Listening Conditions”) was assessed. Participants listened to the stimuli presented in random order and were asked to repeat what they heard. This was done to establish whether performance on the BDS task was affected by the intelligibility of the stimuli.

All testing took part individually in a quiet experimental room at Loughborough University. Participants were seated approximately 70 cm in front of an Apple MacBook Air. For the auditorily presented task, stimuli were delivered through an AudioQuest (California, USA) Dragonfly Red external soundcard and the right earpiece of Sennheiser (Wedemark, Germany) HDA200 headphones, using the open-source audio software Audacity (Version 2.3.3). Consistent with the study of Füllgrabe (2020a), the presentation level for the unprocessed stimuli was set to 70 dB Sound Pressure Level. This corresponds to a raised conversational level which is presumably used by the test administrator when orally presenting stimuli to an older test participant. The same volume setting was used for the listening conditions simulating different levels of severity of HL. For the visually presented tasks, stimuli were displayed in Times New Roman (with a font size of at least 60) on a 13-inch computer screen, using PsychoPy2 (Peirce et al., 2019). At the start of each task, instructions were given verbally by the experimenter. Prior to the administration of the BDS task, participants listened to the stimuli in random order in the simulated-HL condition representing the ARHL of the average 75-year-old person to familiarize them with the degraded stimuli.

The same test format was used for all five memory tasks to facilitate the comparison of performances across tasks. Each task was composed of 14 trials, varying in sequence length from two to eight items to memorize (either digits or letters), with two trials per sequence length. Each task started with a sequence length of two, and then progressed to the next longer sequence. Responses were given verbally by the participants and were scored manually by the experimenter. No feedback as to the correct answer was provided.

Random sequences of digits and letters were generated without replacement using an algorithm implemented in MATLAB (Mathworks, Natick, MA, USA). An additional constraint for digit sequences was that three consecutive digits could not create an easy-to-memorize ascending (e.g., “1-2-3”, “2-4-6”) or descending (e.g., “6-5-4”, “9-7-5”) pattern. Two sets of 14 sequences were created for each memory task and used in a nearly counterbalanced order across participants.

#### Backward-Digit-Span Task and Listening Conditions

Prior to the study, several utterances of each of the digits “1” to “9” were recorded (using a 44.1-kHz sampling rate and 32-bit quantization) from a female native-British speaker with a standard accent. For each digit, the most naturally sounding utterance without artifacts was selected. All retained utterances were equalized in terms of root-mean-square level, before concatenating them and inserting a 1-s-long silence between utterances to create the auditory signals for the BDS task. The task was to recall all digits of a given sequence in reverse order.

**Figure 1:**
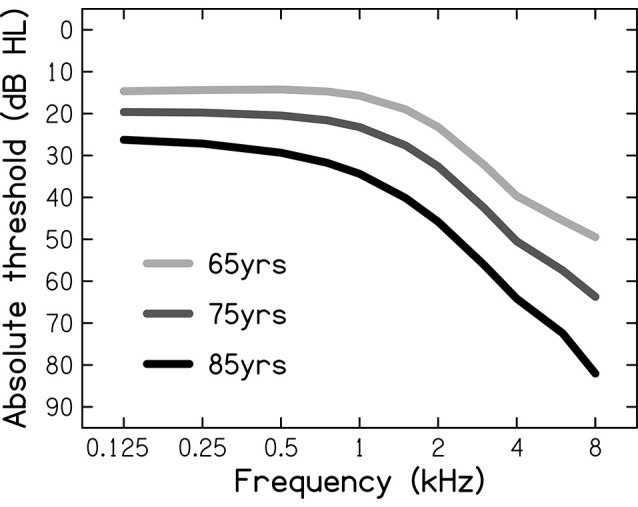
Audiometric thresholds (in dB Hearing Level, dB HL) for the average person aged 65, 75, and 85 years, used as the input for the hearing-loss simulator.

To represent NH, the auditory signals were not further processed. To represent ARHL, the auditory signals were processed through an HL simulator implemented in MATLAB and using an algorithm developed by Nejime and Moore (1997). Based on audiometric thresholds that were used as its input, the HL simulator mimicked some of the perceptual consequences of ARHL: elevated hearing thresholds (by attenuating the frequency components in several frequency bands), reduced frequency selectivity (by spectrally smearing the speech signal; Baer and Moore, 1994), and loudness recruitment (by expanding the range of the speech signal’s envelope; Moore and Glasberg, 1993). In the present study, three different audiograms were used (see Figure 1), representing the hearing sensitivities of the average 65-, 75-, and 85-year-old person, as based on epidemiological audiometric data (Cruickshanks et al., 1998). These audiograms span the range of mild-to-moderate HLs (Stevens et al., 2013).

#### Short-Term-Memory Tasks

The Forward Digit Span (FDS) task (Binet and Simon, 1907; Wechsler, 2008) and the Letter Span (LS) task (Kinsbourne, 1974; Kail and Hall, 2001) are long-established standard measures of STM capacity (Richardson, 2007), in which test participants are required to recall, respectively, sequences of digits (here from 1 to 9) and sequences of letters (here B, F, H, J, L, M, Q, R, and S, based on Norris et al., 2019) in the order in which they were presented. In both tasks, each item to be remembered was displayed on the screen for 1 s, followed by a blank screen for 1 s, before the presentation of the next item.

#### Working-Memory Tasks

The Operation Span (OS) task (Turner and Engle, 1989; Towse et al., 2000) and the Reading Span (RS) task (Daneman and Carpenter, 1980; Füllgrabe and Rosen, 2016) are widely employed different versions of a complex span task, assumed to measure WM capacity (Conway et al., 2005). Both tasks combine a storage component (i.e., the retention of letters and digits for the OS and RS tasks, respectively) with a processing component (i.e., the verification of the results of mathematical operations and the semantic correctness of sentences in the OS and RS tasks, respectively). In a trial, each item to be memorized (letters or digits) was followed by an item to be processed (equations or sentences). At the end of each trial, participants are required to recall the sequence of letters or digits in the order in which they were presented. The equations and sentences, as well as the timing for the presentation of the items, were taken from Stone and Towse (2015).

#### Scoring

The same scoring method as that used by Füllgrabe (2020a) was applied to all tasks: a correct response was awarded when the participant recalled correctly the entire sequence of items to be remembered on a given trial, and the score was weighted by the number of items composing the sequence (e.g., correctly recalling all items of a six-item sequence earns a score of 6 while recalling only five items of that sequence earns a score of 0). As all tasks used the same number of trials and the same sequence lengths, the maximum score in all tasks was 70.

### Statistical Analysis

All statistical analyses were conducted using SPSS 24 (IBM Corp., Armonk, NY, USA). As a Shapiro-Wilk test revealed that BDS scores for the simulated-HL conditions for the average 75- and 85-year-old person were not normally distributed, non-parametric tests were used to assess the significance of the effect of simulated HL. Differences between listening conditions were assessed using a Friedman test, followed by one-tailed Wilcoxon signed-rank tests. Spearman’s rank correlation coefficient was computed to analyze the association between BDS scores and scores obtained on the STM and WM tasks. The significance of the difference between correlation coefficients was assessed based on the two-tailed test described by Lee and Preacher (2013). In the case of multiple comparisons, uncorrected test results are reported, but their significance was confirmed against Holm-Bonferroni corrected significance levels. For all tests, the criterion used for statistical significance was *p* < 0.05.

## Results

### Intelligibility of Processed Stimuli

All participants were able to identify all nine digits processed to mimic ARHL experienced by the average 85-year-old person. Hence, it can be assumed that the intelligibility of the digits, even though only assessed in the most severe simulated-HL condition, was also perfect (i.e., 100% correct) in the milder simulated-HL conditions in which the BDS task was conducted.

### Effect of Simulated Hearing Loss on BDS Scores

**Figure 2:**
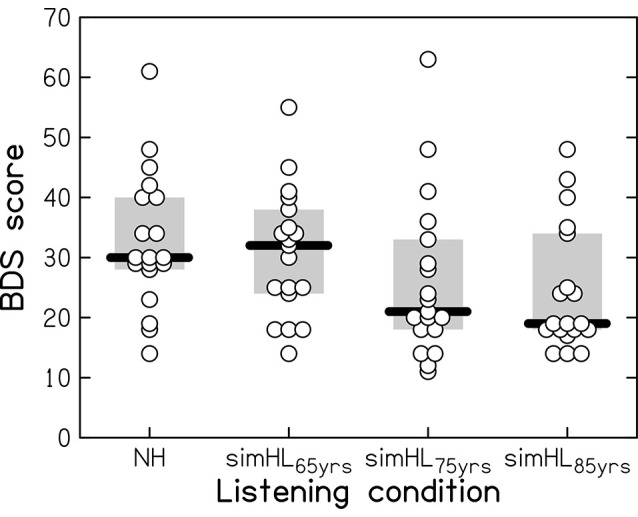
Backward-Digit-Span(BDS) performance (with a maximum score of 70) as a function of listening condition: in the case of normal-hearing (NH) and with three progressively more severe simulated hearing losses, associated with the hearing sensitivity of the average 65-, 75-, and 85-year-old person (simHL_65yrs_, simHL_75yrs_, and simHL_85yrs_, respectively). Horizontal thick bars indicate the median, the light-gray-shaded boxes represent the interquartile range, and the open circles denote individual data points. Overlapping data points are displaced horizontally for better visibility.

Performance on the BDS task is shown in Figure 2 for the four listening conditions: the normal hearing (NH) and the simulated-HL conditions (simHL_65yrs_, simHL_75yrs_, and simHL_85yrs_). The variability of the data was large, even in the unprocessed listening condition. Compared to the NH condition, the simHL_65yrs_ condition yielded similar median BDS performance, while the simulated-HL conditions for the average 75- and 85-year-old person yielded markedly lower median scores (by 9 and 11 points, respectively). Friedman’s analysis of variance confirmed that BDS performance differed across listening conditions (*χ*^2^_(3)_ = 17.674, *p* < 0.001). While performance in the simHL_65yrs_ condition was not significantly different from that obtained in the NH condition (*z* = −1.089, *p* = 0.145), the observed declines in the simHL_75yrs_ (*z* = −2.789, *p* = 0.002) and simHL_85yrs_ (*z* = −2.943, *p* = 0.001) conditions were significant. There was no significant difference in BDS performance between the two most severe simulated-HL conditions (*z* = −1.166, *p* = 0.128).

### Association Between Performances on the STM and WM Tasks

Results for the four visually presented memory tasks are shown in Supplementary Figure 1. Performances on the two simple-span tasks (FDS and LS tasks) and performances on the two complex-span tasks (OP and RS tasks) respectively correlated strongly and very strongly (ρ = 0.70, *p* < 0.001 for the STM tasks; ρ = 0.81, *p* < 0.001 for the WM tasks). To obtain a more representative and stable estimate of STM and WM capacity for each participant, scores from the two STM tasks and scores from the two WM tasks, respectively, were averaged. The correlation between mean estimates for simple- and complex-span tasks was moderate (ρ = 0.50, *p* = 0.014).

### Association Between Performances on the BDS Task and the STM and WM Tasks

Since simulating the perceptual consequences of ARHL for the average 65-year-old person did not affect median BDS performance (see Figure 2), and to reduce the number of statistical tests conducted for the relatively small sample size used in the present study, the association of BDS scores with mean STM and WM scores was investigated only for the two “extreme” listening conditions (i.e., the NH and simHL_85yrs_ conditions; see Table 1).

**Table 1:** Spearman’s rank correlation coefficients for the relationship between listening condition (NH and simHL_85yrs_) for the BDS task and the two other types of memory tasks (STM and WM).

BDS listening condition	STM	WM
NH	0.66**	0.50*
simHL_85yrs_	0.32	0.77***

*p < 0.05; **p < 0.01; ***p < 0.001.

In the NH condition, BDS scores correlated significantly with STM (ρ = 0.66, *p* = 0.001) and WM scores (ρ = 0.50, *p* = 0.014); the positive correlation was strong and moderate, respectively. However, the two correlation coefficients did not differ significantly from each other (*z* = −0.864, *p* = 0.387). In the simHL_85yrs_ condition, there was a significant strong positive correlation between BDS and WM scores (ρ = 0.77, *p* < 0.001), but the association of BDS scores with STM scores was weak and nonsignificant (ρ = 0.32, *p* = 0.088); the correlation coefficient involving WM scores was significantly stronger than that involving STM scores (*z* = 2.458, *p* = 0.014).

## Discussion

The importance of cognition for speech intelligibility seems widely acknowledged in the HHC sector (Rembaud et al., 2017), and there is an increasing call for the use of cognitive tests in audiological practice (Shen et al., 2016; American Speech-Language-Hearing Association, 2018). However, relatively few HHC professionals currently conduct additional cognitive assessments (Rembaud et al., 2017; Raymond et al., 2020). This is possibly the consequence of a lack of clear guidelines as to who should be screened (e.g., the age above which the assessment should be conducted) and the absence of appropriate tests for the screening of patients with sensory impairments. Interestingly, while the awareness that sensory processing abilities can affect cognitive functioning is not new (Rabbitt, 1990; van Boxtel et al., 2000), the distinction between lower cognitive-test performance due to assessment-format-related factors and lower performance due to permanent neuroplastic changes following sensory deprivation is rarely being made, even though both are likely to occur in older people with ARHL.

To investigate in isolation the immediate sensory biases occurring during cognitive assessment (and that could be mitigated by adjusted test-administration methods; Dupuis et al., 2015; Shen et al., 2020; Davis, 2021), some studies have adopted an impairment-simulation approach with NH persons (Lindenberger et al., 2001; Jorgensen et al., 2016; Gaeta et al., 2019). Given the decreasing prevalence of audiometrically NH adults with increasing age (Cruickshanks et al., 1998), it can be challenging to recruit a sufficiently large number of participants from this population (Füllgrabe et al., 2015). Therefore, young NH participants were often used, based on the assumption that they constitute a valid model of older NH listeners. As regards the supra-threshold processing deficits implemented in the HL simulator used in the present study, they seem only slightly affected by aging in the absence of elevated audiometric thresholds (Peters and Moore, 1992; Sommers and Humes, 1993; Gifford and Bacon, 2005). Hence, young and older NH participants are presumably impacted in similar ways by the HL simulation used in the present study.

The aim of the present study was to confirm the findings of a previous HL-simulation study, using a more comprehensive simulation of ARHL than the loss of audibility, and a robust experimental design. Consistent with results reported by Füllgrabe (2020a), BDS scores were significantly lower in the simulated HL conditions mimicking the perceptual consequences of HL of the average 75-year-old person and older than in the NH condition. The reduction in performance was not due to compromised intelligibility of the test stimuli. Hence, while for severe cases of HL, the ability to hear the test stimuli is likely the main (and possibly a sufficient) factor for impaired performance on auditory-based cognitive assessments, it is not a necessary condition, as cognitive performance may be affected even when intelligibility is perfect (Nittrouer and Lowenstein, 2014; Füllgrabe, 2020a). This could be explained by the existence of age- and HL-related deficits in supra-threshold auditory processing abilities (Füllgrabe and Moore, 2018; Ozmeral et al., 2018; Anderson and Karawani, 2020) which have been shown to be associated with speech identification (Lorenzi et al., 2006; Bernstein et al., 2013; Füllgrabe et al., 2015). In case of reduced intelligibility and/or supra-threshold auditory processing abilities, lower cognitive-test performance could be due to additional perceptual efforts being required to achieve speech understanding, thereby reducing the amount of cognitive resource available for the execution of the cognitive task itself (Rabbitt, 1991; Wingfield et al., 2005).

No significant decline in performance was observed for the mildest ARHL simulated in the present study. This could be interpreted as indicating that the cognitive-test performance of persons aged below 75 years is not affected by ARHL. However, performance on the Hopkins Verbal Learning Test, a different memory task requiring the immediate verbal recall of lists of words, has been shown to be significantly reduced by simulated HL representative of a person as young as 70 years (Füllgrabe, under revision). Hence, while establishing a cutoff below which cognitive-test performance is not affected would certainly be desirable for clinicians and researchers alike, it is likely that its exact value depends on the specific cognitive task being used.

There was no further reduction in memory performance when simulating the most severe level of ARHL used in the present study (corresponding to a moderate HL). This was possibly due to the apparently high difficulty level of the BDS task, as floor effects were observed for some of the participants already for the milder simulated-HL conditions.

Previously (Füllgrabe, 2020a, under revision) and in the present study, the different simulated-HL conditions were defined relative to age-group-specific epidemiological audiometric data (Cruickshanks et al., 1998). This was done with clinicians and researchers in mind who only have access to the test person’s age but not their hearing sensitivity. However, given that these age-referenced listening conditions rely on average audiometric data, predicting an auditory bias for individual test participants based on their chronological age is only approximate. To derive clinical recommendations as to which individual might be at risk of being cognitively mis-assessed, a more appropriate approach would be to investigate the impact on cognitive processing and performance of different levels of HL severity defined by audiometric boundaries and audiometric shape (Bisgaard et al., 2010; Cruickshanks et al., 2020).

After averaging performances within each type of memory task (i.e., simple vs. complex measures), mean STM and WM performances correlated only moderately, consistent with the notion that either partly different subcomponent processes are at play when completing the two tasks (as observed in children; Kail and Hall, 2001), or that the same subcomponent processes are used to different extents (Unsworth and Engle, 2007). Performance on the BDS task presented in the unprocessed listening condition correlated moderately and similarly with STM and WM performances, indicating that cognitive processes required for the two visually presented types of memory tasks are also used by NH persons when completing an auditory version of the BDS task. On the other hand, performance on the BDS task presented in the most severe HL condition simulated here (i.e., the simHL_85yrs_ condition) was only (but strongly) associated with performance on the WM tasks. In comparison, St Clair-Thompson (2010), using only visually presented memory tasks, found in young adults that performance on the BDS task was more closely related to measures of STM than to measures of WM. In children, however, the opposite trend was observed. This developmental effect was explained by children employing not only storage but also executive-attentional resources for the digit recall in reverse order (Elliot et al., 1997), while, for adults, the tasks is less attentionally demanding and mainly draws on coding and rehearsal processes (Rosen and Engle, 1997). Applying the same reasoning to the present study, it can be speculated that the completion of the BDS tasks required the involvement of additional executive-attentional resources in the moderate simulated-HL condition compared to the NH condition.

The finding of a strong association between BDS scores and scores on the WM tasks in the presence of simulated HL also has practical implications for the joined administration of the FDS and BDS tasks (e.g., as part of the same subtest of the Wechsler Intelligence Scales) to people with HL. Given that the recall in reverse order of sequences of digits is more demanding on WM under HL, BDS performance should probably not be combined with FDS performance into a single score when people with HL are tested, as the two tasks are not tapping the same cognitive processes.

### Study Limitations

Several additional caveats regarding the reported findings should be noted:

A relatively small sample size was used in the present study. Nevertheless, the study’s main finding of a significant effect of simulated HL on cognitive-test performance is at least qualitatively consistent with results from previous simulation studies (Jorgensen et al., 2016; Wong et al., 2019; Füllgrabe, 2020a, under revision). In contrast, the conclusion drawn from the correlational analyses that different cognitive processes may be at play during the execution of the BDS task by adults with and without ARHL needs to be considered with caution until a replication of the results is reported for a larger sample.

Participants were not given any practice on the cognitive tasks prior to their administration. This might explain the large interindividual variability in memory performance and floor effects in some of the simulated-HL conditions. The provision of training items would probably reduce any procedural difficulties with the task but is generally not included in clinical cognitive assessments (e.g., Wechsler Adult Intelligence Scale; Wechsler, 2008).

The HL simulator only mimicked some of the perceptual consequences of ARHL. Other auditory processing deficits (e.g., a reduction in sensitivity to temporal cues; Füllgrabe, 2013; Wallaert et al., 2016) related to age- and HL-related changes (e.g., synaptopathy, reduced function of the stria vascularis; Liberman and Kujawa, 2017; Heeringa and Köppl, 2019) were not simulated. Thus, the true size of the auditory bias in cognitive assessment is probably larger than that reported here.

Only the effect of simulated HL on a single cognitive test that requires the processing of auditorily presented stimuli was investigated. Intuitively, an auditory bias would not be expected for cognitive tasks using test stimuli that are presented in other sensory modalities (e.g., visual stimuli), and thus the current findings are only applicable to a subset of cognitive tasks. However, in most cognitive assessments, the presentation of the aim and procedure of the task, as well as specific test instructions, are given orally. Since HL affects the comprehension of speech in general and of instructions in particular (Henn et al., 2017), it is possible that, independently of the presentation format of the test stimuli, cognitive-test performance is affected by HL.

## Conclusions

The cognitive processes involved in the completion of the auditorily presented BDS task and the performance on this task are affected by the simulated (and presumably actual) hearing abilities of the test participant. Ensuring good intelligibility of the test stimuli may not eliminate this bias. This calls into question the validity of the assumption that cognitive assessments provide a sensory-bias-free and process-stable estimate of cognitive functioning. In the case of auditory cognitive tasks, the hearing abilities of the test participants need to be considered when interpreting the cognitive underpinnings of and the performance on the task in order to avoid the mischaracterization of cognitive functioning (Füllgrabe, 2020a, b).

## Data Availability Statement

The dataset analyzed for this study can be obtained from the corresponding author for any research purpose.

## Ethics Statement

The study was approved by the Loughborough University Ethics Approvals (Human Participants) sub-committee (reference number: UG723). Informed written consent was obtained from all participants involved in the study.

## Author Contributions

CF: conceptualization, formal analysis, writing—original draft preparation, writing—review and editing, visualization, supervision, and project administration. CF and OÖ: methodology, validation, resources, and data curation. OÖ: software and investigation. All authors contributed to the article and approved the submitted version.

## Supplementary Material

Supplementary Figure 1 Performance (with a maximum score of 70) for the two short-term-memory tasks (Forward Digit Span, FDS; Letter Span, LS) and the two working-memory tasks (Operation Span, OS; Reading Span, RS). Horizontal thick bars indicate the median, the light-gray-shaded boxes represent the interquartile range, and the open circles denote individual data points. Overlapping data points are displaced horizontally for better visibility.
