# Supplementary figures and images for: Immediate Effects of (Simulated) Age-Related Hearing Loss on Cognitive Processing and Performance for the Backward-Digit-Span Task
Source: Front Aging Neurosci. 2022 Oct 13;14:912746. doi: 10.3389/fnagi.2022.912746 (PMC9677092; doi:10.3389/fnagi.2022.912746)

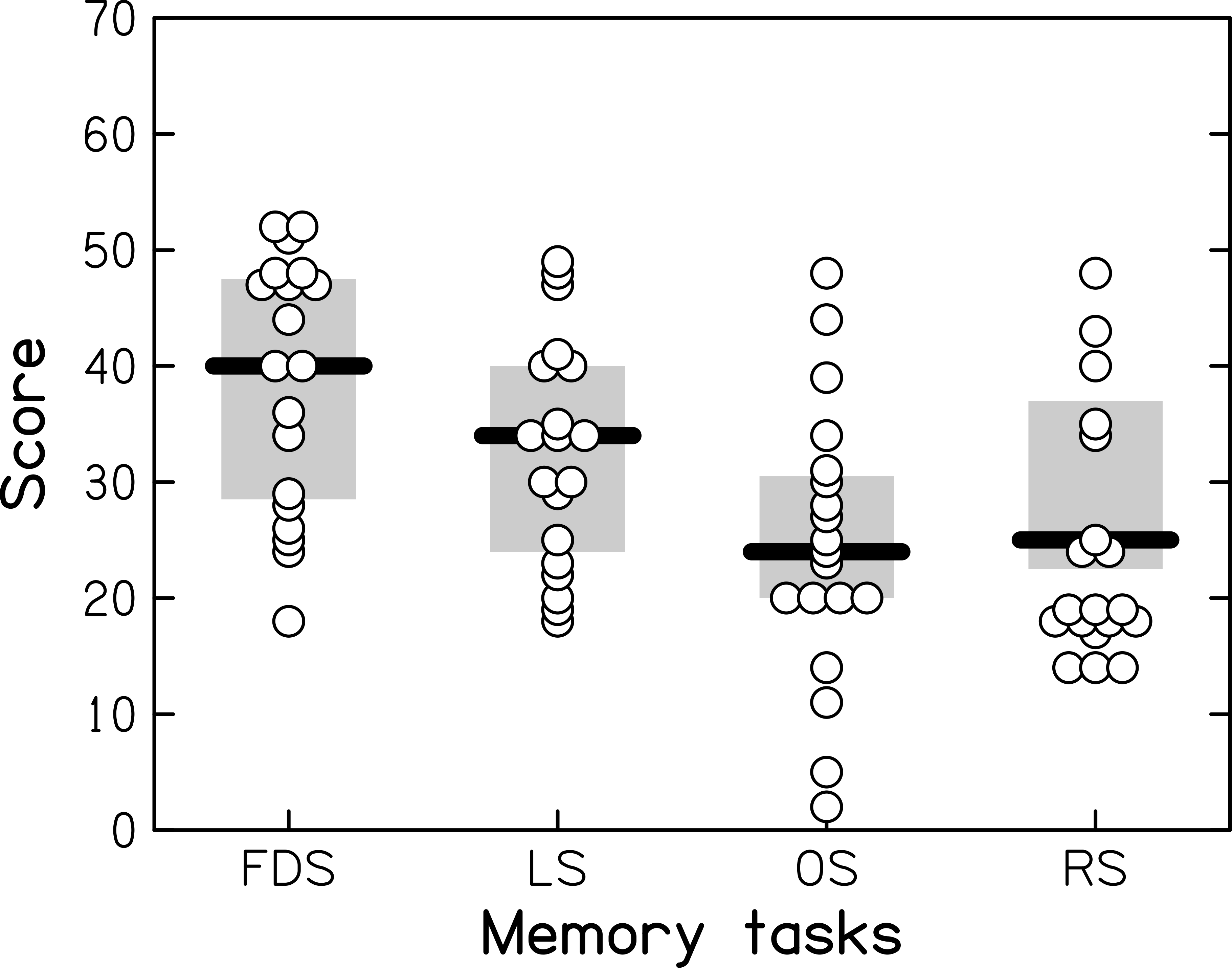

Supplement: Supplementary Figure 1 — Performance (with a maximum score of 70) for the two short-term-memory tasks (Forward Digit Span, FDS; Letter Span, LS) and the two working-memory tasks (Operation Span, OS; Reading Span, RS). Horizontal thick bars indicate the median, the light-gray-shaded boxes represent the interquartile range, and the open circles denote individual data points. Overlapping data points are displaced horizontally for better visibility. [file Image_1.tif]
